# Activation of the B Cell Receptor Leads to Increased Membrane Proximity of the Igα Cytoplasmic Domain

**DOI:** 10.1371/journal.pone.0079148

**Published:** 2013-11-11

**Authors:** Wing-Yiu Lee, Pavel Tolar

**Affiliations:** Division of Immune Cell Biology, National Institute for Medical Research, London, United Kingdom; University of New South Wales, Australia

## Abstract

Binding of antigen to the B cell receptor (BCR) induces conformational changes in BCR's cytoplasmic domains that are concomitant with phosphorylation of the immunoreceptor tyrosine-based activation motifs (ITAMs). Recently, reversible folding of the CD3ε and ξ chain ITAMs into the plasma membrane has been suggested to regulate T cell receptor signaling. Here we show that the Igα and Igβ cytoplasmic domains of the BCR do not associate with plasma membrane in resting B cells. However, antigen binding and ITAM phosphorylation specifically increased membrane proximity of Igα, but not Igβ. Thus, BCR activation is accompanied by asymmetric conformational changes, possibly promoting the binding of Igα and Igβ to differently localized signaling complexes.

## Introduction

The B cell receptor (BCR) provides signals for the development, activation and differentiation of B lymphocytes. Expression of the BCR on the cell surface requires assembly of the membrane-bound immunoglobulin (mIg) with the heterodimer of Igα and Igβ [1],[2] in a 1∶1 stoichiometry [3],[4]. The association of the mIg with Igαβ is required for all tonic and antigen-induced intracellular signaling. Igα and Igβ are covalently linked via a disulfide bond in the extracellular domains [5],[6] and the heterodimer associates non-covalently with the mIg via the transmembrane and extracellular domains [3],[6]–[8]. The cytoplasmic tails of Igα and Igβ each contain an immunoreceptor tyrosine-based activation motif (ITAM). The ITAM consists of two precisely spaced tyrosines each followed by a hydrophobic residue at position +2. Upon antigen binding, Src-family kinases phosphorylate tyrosine residues in ITAMs, leading to the recruitment of the tyrosine kinase Syk. Although cytoplasmic domains of both Igα and Igβ contain ITAM motifs, they serve non-redundant functions in B cell development and differentiation [9]–[15]. These non-redundant functions have been attributed to binding of different signaling proteins to the Igα and Igβ cytoplasmic domains [16], either due to different non-tyrosine residues within the ITAMs [17] or due to the presence of non-ITAM tyrosines in Igα [14],[18].

Florescence resonance energy transfer (FRET) imaging showed that antigen binding quickly clustered the BCR and then lead to a reversible increase in the distances between the cytoplasmic domains, suggesting that BCR clusters undergo ‘opening’ at the cytoplasmic side [4],[19]. Opening of preformed BCR clusters has also been suggested to activate signaling, based on fluorescence complementation techniques [20]. These results suggested that recruitment of signaling proteins to the Igα and Igβ may be regulated by geometric constraints arising from the order of the cytoplasmic domains. However, the exact configuration of cytoplasmic domains of Igα and Igβ in resting and activated cells is not well understood.

Recently, there has been accumulation of evidence that the cytoplasmic domains of two components of the T cell receptor (TCR), TCRζ and CD3ε, reversibly fold into helical structures that bind to negatively charged phospholipid membranes [21],[22]. The structure of the CD3ε ITAM bound to phospholipid bicelles showed that this binding buries the ITAM tyrosines into the hydrophobic core of the membrane bilayer [22]. Thus, access to the ITAMs during T cell activation could be regulated by mechanisms that release the cytoplasmic domains from the plasma membrane [23],[24]. The interaction of the CD3ε cytoplasmic domain with the plasma membrane depends on stretches of positive residues preceding the ITAM. While hydrophobicity is a universal feature of ITAMs, the presence of positively charged residues is variable amongst immunoreceptors. The extent to which other immunoreceptors' cytoplasmic domains interact with the plasma membrane thus remains to be experimentally determined.

Here we used FRET in live B cells to measure the proximity of BCR cytoplasmic domains to the plasma membrane in the resting state and upon antigen binding. We show that while the cytoplasmic domains of Igα and Igβ did not associate intimately with the plasma membrane in resting B cells, the proximity of Igα cytoplasmic domain to plasma membrane increased upon BCR engagement. This change in membrane proximity was intrinsic to the Igα cytoplasmic domain and depended on the phosphorylation of the ITAM tyrosines by a Src-family kinase.

## Results And Discussion

**Figure 1:**
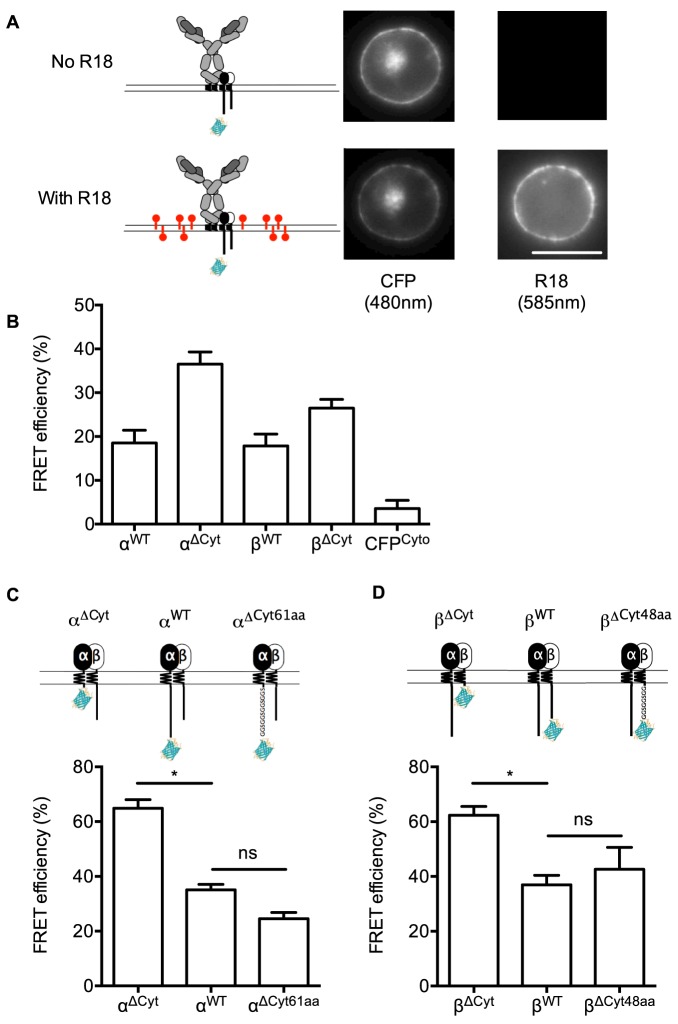
Measurement of the proximity of BCR cytoplasmic domains to the plasma membrane. (**A**) Left, diagram of FRET measurement by quenching of CFP by the membrane dye R18. Right, images of HEK293A cells expressing BCR with CFP-tagged IgαΔ^Cyt^ before and after incubation with R18. Scale bar, 10 µm. (**B**) FRET efficiency measured by quenching of CFP attached to the indicated constructs in HEK293 cells. WT, wild type, ΔCyt, truncation of the cytoplasmic domain, CFP^cyto^, cytoplasmic CFP. Data represent mean and s.e.m. of n = 5–13 cells from at least 3 experiments. (**C, D**) FRET measurement in primary B cells. Schematic depiction of Igα (**C**) and Igβ (**D**) constructs and their corresponding FRET efficiency measured as in (A). All constructs associated with endogenous mIg (not depicted). ΔCyt61aa and ΔCyt48aa are constructs where the cytoplasmic domains were replaced by flexible linkers. Data represent mean and s.e.m. of n = 5–15 cells from at least 3 experiments. *, p<0.05 in Mann-Whitney tests, ns, not significant.

To investigate the relationship of the cytoplasmic domains of Igα and Igβ to the plasma membrane in unstimulated cells, we used FRET to measure proximity between cyan fluorescent protein (CFP) attached to the C-termini of Igα and Igβ constructs, and the lipophilic dye octadecyl rhodamine B chloride (R18), which incorporates into the plasma membrane. To set up FRET experiments, we transfected HEK293T cells with constructs of Igα and Igβ, together with IgM heavy chain and Igλ light chain, which resulted in expression of the BCR constructs at the plasma membrane. To determine FRET efficiency, we measured quenching of the FRET donor, CFP, at the plasma membrane, by incorporation of the FRET acceptor, R18 (Fig. 1A, B). Control experiments with cells expressing CFP in the cytoplasm (CFP^Cyto^) showed little FRET as expected, while attachment of CFP to the C-termini of cytoplasmic domain-truncated mutants (ΔCyt) of either Igα or Igβ resulted in ∼28–35% FRET (Fig. 1B). Wild-type Igα and Igβ constructs had ∼18% FRET, showing that their C-termini are at a distance from the plasma membrane that is substantially longer than that of the ΔCyt constructs.

Similar results were obtained after transfection of the Igα and Igβ constructs into primary B cells, where they incorporated into endogenous BCR complexes at the cell surface (Fig 1C, D). To understand how the specific sequences of the cytoplasmic domains of Igα or Igβ contributed to the distance of their C-termini from the plasma membrane, we replaced the cytoplasmic domains in Igα and Igβ with hydrophilic flexible linkers of lengths identical to the wild type proteins (ΔCyt61aa and ΔCyt48aa constructs). Measurements in primary B cells showed that FRET levels of the linker constructs were indistinguishable from the wild-type constructs (Fig 1C, D), indicating that Igα and Igβ do not maintain structures that would bring the C-termini into close proximity of the plasma membrane.

**Figure 2:**
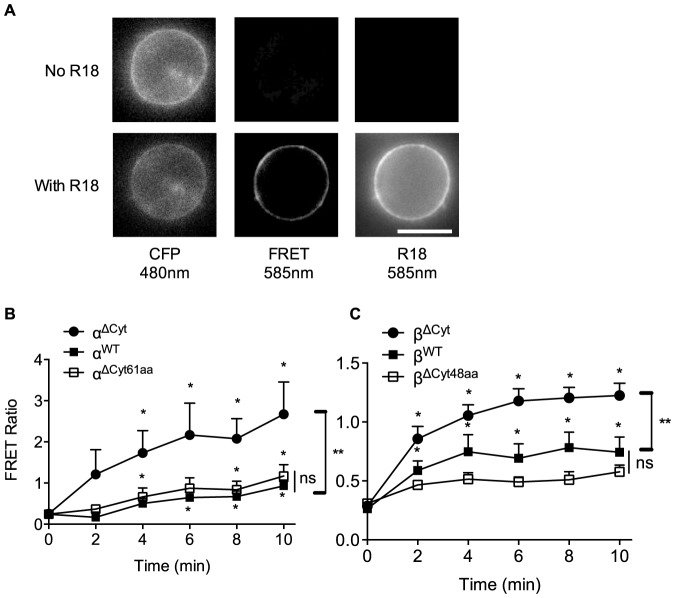
Dynamic measurement of the distance of the BCR cytoplasmic domains from the plasma membrane by ratiometric FRET in primary B cells. (A) Images of a B cell expressing CFP-tagged Igα showing the CFP, FRET and R18 channels before and after incubation with R18. Scale bar, 10 µm. (B, C) Timelapse of FRET ratios of the indicated constructs during incubation with R18. Data represent mean and s.e.m., n = 5–15 cells from at least 3 experiments. *, p<0.05 in Wilcoxon paired tests of FRET ratios of the indicated timepoints against t = 0 of the corresponding construct. **, p<0.05 in Mann-Whitney tests of FRET ratios comparing the indicated constructs at t = 10. Ns, not significant.

**Figure 3:**
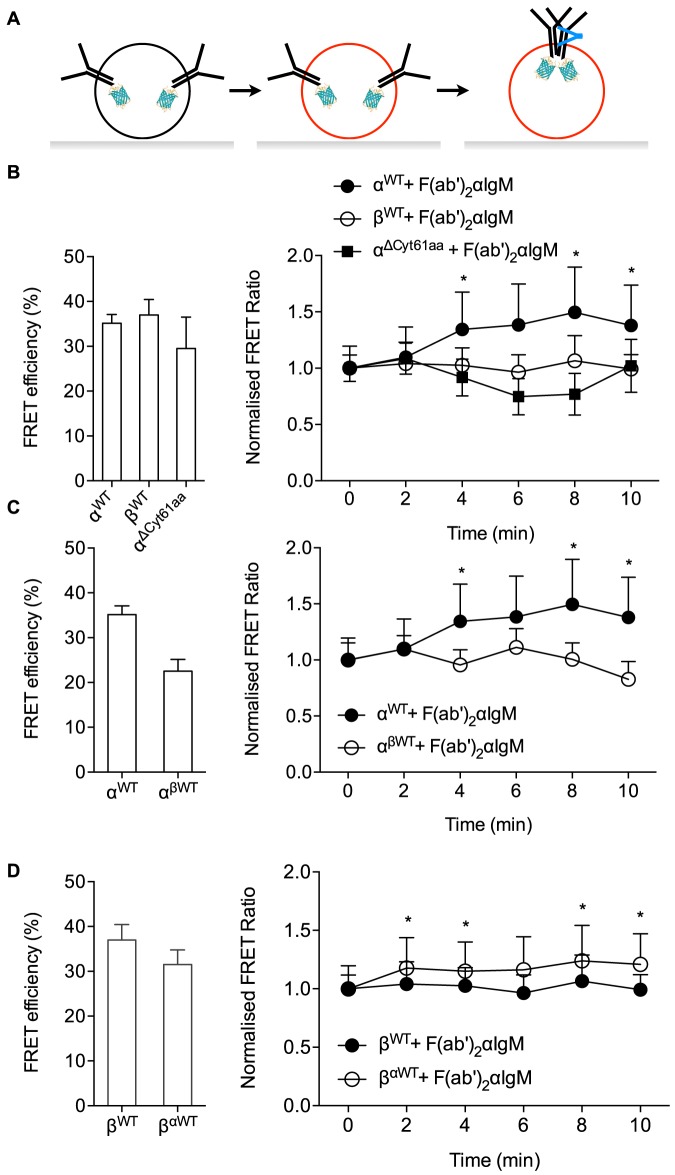
BCR activation increases membrane proximity of the cytoplasmic domain of Igα, but not of Igβ. (**A**) Schematic diagram of ratiometric FRET measurement in B cells stimulated with anti-IgM. (**B, C, D**) Left, FRET efficiency determined by CFP quenching in resting B cells. Right, FRET ratios normalized to FRET ratios in resting cells after stimulation of the cells with anti-IgM for the indicated times. Data represent mean and s.e.m., n = 9–16 cells from 3 experiments. *, p<0.05 in Wilcoxon paired test for FRET ratios of individual timepoints against t = 0 of the corresponding constructs. Data for Igα^WT^ and Igβ^WT^ from (B) are replotted in (C) and (D), respectively, for comparison.

To understand how membrane proximity of Igα and Igβ cytoplasmic domains changes after antigen binding, we used dynamic timelapse imaging of sensitized acceptor emission (Fig. 2A). We first monitored changes in FRET ratios upon addition of R18. As expected, FRET ratios increased gradually during R18 incorporation in cells expressing the Igα or Igβ constructs (Fig 2B, C). In agreement with FRET efficiency determined by CFP quenching, the final FRET ratios in cells expressing wild-type Igα (Fig. 2B) or Igβ (Fig. 2C) were significantly lower than FRET ratios in cells expressing the ΔCyt constructs, but they were similar to the corresponding linker constructs, ΔCyt61aa and ΔCyt48aa, respectively. To monitor changes in proximity of Igα and Igβ cytoplasmic domains to the plasma membrane after antigen binding, we imaged R18-loaded B cells before and after stimulation with antigen (Fig 3A). Measurements of CFP quenching during R18 loading confirmed that the levels of FRET in resting cells were similar to those described above (Fig 3B C, D, left panels). We then normalized the FRET ratio to the FRET ratio of R18 loaded, unstimulated cells. Antigen stimulation caused a significant increase in the FRET ratio of the wild-type Igα construct, whereas the FRET ratio of the Igβ construct remained similar to resting cells (Fig. 3B). This result suggests that BCR activation increases the proximity of the cytoplasmic domain of Igα, but not of Igβ, to the plasma membrane.

The antigen-induced proximity of Igα to the plasma membrane depended on the sequence of the intracellular tail of Igα as antigen stimulation did not increase membrane proximity of the Igα^ΔCyt61a^ construct (Fig. 3B). To understand if the induced proximity depended on the extracellular and transmembrane domains of Igα, we measured FRET ratios in B cells expressing wild-type Igα or a construct, in which Igα cytoplasmic domain was replaced with Igβ cytoplasmic domain (α^βWT^). This swapped construct showed no change in FRET ratio upon antigen binding (Fig. 3C). Conversely, a construct of Igβ in which the cytoplasmic domain was replaced with the cytoplasmic domain of Igα (β^αWT^) showed an increase in FRET ratio upon antigen binding (Fig. 3D), although to a slightly lower degree than the wild-type Igα. These results show that the increase in plasma membrane proximity of Igα upon BCR activation is intrinsic to the cytoplasmic domain of Igα. The less prominent increase in membrane proximity of the βαWT swap construct could be due to less efficient signaling from the BCR containing only Igα intracellular domains.

**Figure 4:**
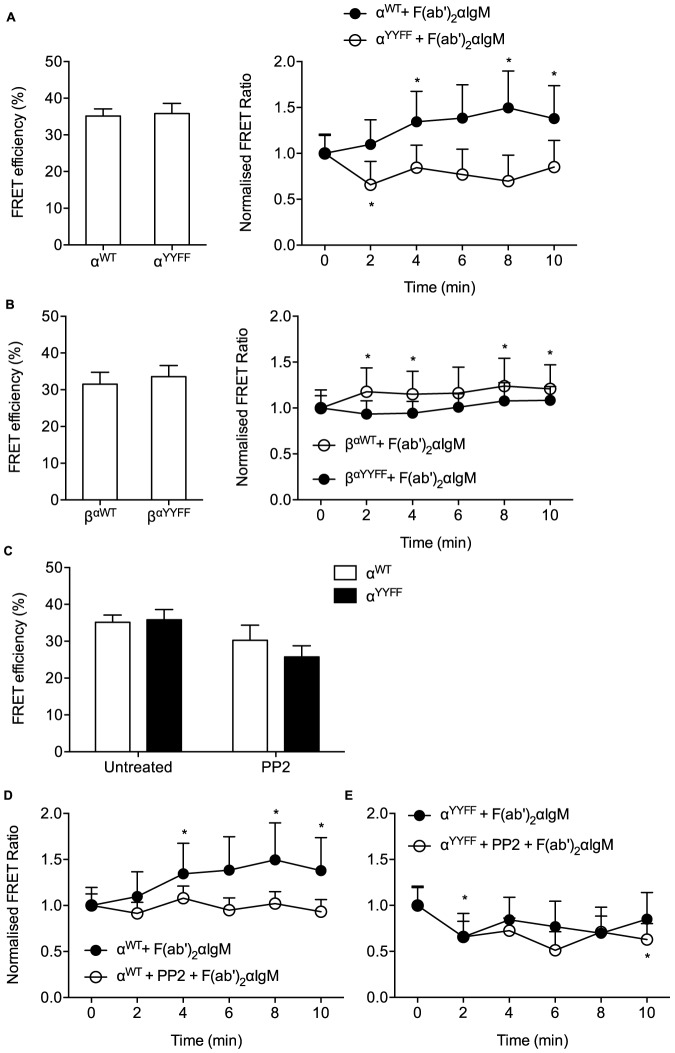
Phosphorylation of Igα ITAM tyrosines is required for the increased membrane proximity of Igα cytoplasmic domain upon BCR activation. (A, B) Increased membrane proximity requires ITAM tyrosines. Left, FRET efficiency determined by CFP quenching in resting B cells. Right, normalized FRET ratios during stimulation of the cells with anti-IgM. Data represent mean and s.e.m. of 10–19 cells from 3 experiments. (C, D, E) Increased membrane proximity requires Src-family kinase activity. (C) FRET efficiency determined by CFP quenching in resting B cells in the presence or absence of PP2. (D, E) Normalized FRET ratios during stimulation of the cells with anti-IgM in the presence or absence of PP2. Data represent mean and s.e.m. of 14–16 cells from 3 experiments. *, p<0.05 in Wilcoxon paired test for FRET ratios of individual timepoints against t = 0 of the corresponding constructs. Data for Igα^WT^ and for Igβα^WT^ are replotted from Fig. 3, data for Igα^YYFF^ in (A) are replotted in (E) for comparison.

To understand if phosphorylation of the Igα ITAM is required for the change in membrane proximity we mutated ITAM tyrosines to phenylalanines (α^YY/FF^). This mutation did not affect the level of FRET in resting cells (Fig. 4A, left panel), however, it abolished the increase in FRET ratio upon antigen binding (Fig. 4A, right panel) and resulted even with a modestly decreased FRET ratio. Similarly, mutations of ITAM tyrosines in the cytoplasmic domain of the β^αWT^ swap construct (β^αYY/FF^) abolished the increase of FRET ratio upon antigen binding without affecting the basal FRET efficiency (Fig. 4B). To investigate if Src-family kinase activity is required for the increase in plasma membrane proximity of Igα cytoplasmic domain upon BCR activation, we treated the transfected B cells with a Src-family kinase inhibitor, PP2. PP2 did not affect the basal FRET efficiency in B cells expressing wild-type or YY/FF Igα (Fig. 4C). However, PP2 abolished the antigen-induced increase in FRET efficiency in wild-type Igα (Fig. 4D), while not affecting the FRET efficiency of the α^YY/FF^construct (Fig. 4E). Thus, Src-family kinase-mediated phosphorylation of ITAM tyrosines in Igα is required for the increased plasma membrane proximity of Igα cytoplasmic domain upon BCR activation. In contrast, in the absence of ITAM tyrosines, antigen binding to the BCR resulted in a slightly increased distance of the Igα C-terminus from the plasma membrane, possibly due to the geometric constraints resulting from BCR clustering.

So far the only ITAMs shown to associate with membranes are the TCRζ and CD3ε. In CD3ε, plasma membrane binding required a cluster of positively charged residues between the transmembrane domain and the ITAM [22]. The function of the positive charge is likely to mediate binding to the negatively charged phosphatidylserine in the inner leaflet of the plasma membrane. Neither Igα, or Igβ have large clusters of positively charged residues, and we did not detect significant difference in membrane proximity between Igα or Igβ, and constructs, in which the cytoplasmic tails were replaced by a sequence expected to form a random coil. We conclude that in resting B cells, Igα and Igβ ITAMs do not associate intimately with the plasma membrane.

However, upon BCR stimulation, we observed that while Igβ cytoplasmic domain remained at a constant distance from the plasma membrane, Igα cytoplasmic domain moved closer. This increased membrane proximity was intrinsic to the Igα cytoplasmic tail and depended on tyrosine phosphorylation of the Igα ITAM. These findings suggest that the movement of Igα towards the plasma membrane contributes to the previously reported ‘opening’ of the BCR cytoplasmic domains [4], and to the increase in FRET of Igα with membrane probes enriched in lipid raft fractions of the plasma membrane [19],[25]. As the phosphorylated ITAMs cannot directly fold into the plasma membrane [21],[22], our data suggest that the movement of the Igα cytoplasmic domain is mediated by inducible binding to a membrane-associated signaling molecule, downstream of ITAM phosphorylation. One candidate for such binding are Src-family kinases themselves [26]. Alternatively, it is possible that Igα participates in assembly of plasma membrane complexes that regulate phospholipid metabolism, which plays a prominent role in both positive and negative regulation of BCR signaling. For example, Igα contains a non-ITAM tyrosine, which is phosphorylated after antigen binding downstream of ITAM phosphorylation and recruits the adaptor protein BLNK [18],[27]. BLNK binds phospholipase Cγ, which requires interaction with inner leaflet of the plasma membrane for its enzymatic activity.

Our results are thus consistent with the view that Igα and Igβ are differentially phosphorylated and recruit distinct effector molecules [28],[29]. As result, Igα and Igβ have non-overlapping functions in B cell development and activation [9]–[15]; some of these functions may depend on the regulation of phospholipid signaling.

## Materials And Methods

All experiments were approved by the ethical review panel at the National Institute for Medical Research and conducted under British Home Office regulations (project license PPL 80/2506).

### Mice And Cells

C57/BL6 mice were bred in the National Institute for Medical Research under SPF conditions. Primary naïve B cells were obtained from splenocytes following red blood cell lysis and negative selection with anti-CD43 microbeads (Miltenyl biotec). Purified B cells were cultured at 5x106/ml for 24 hours in RPMI1640 supplemented with 10% FCS, 2mM Glutamine, 50 µM β-mercaptoethanol (Invitrogen) and 1 µg/ml CpG (5′-TCCATGACGTTCCTGACGTT-3′, Sigma) prior to transfection. HEK293A cells (Quantum Biotechnologies) were maintained at 1×106/ml in Pro293S medium (Lonza) supplemented with 1.5% FCS and 2 mM Glutamine, using agitation at120 rpm.

### Constructs And Transfections

The monomeric version of CFP was attached to the C-termini of mouse Igα and Igβ. In-Fusion HD cloning kit (Clontech) was used to create mutants with deletion of cytoplasmic domains (ΔCyt) in Igα (amino acids 160 – 220) and in Igβ (amino acids 181 – 228), mutants containing substitution of cytoplasmic domains with corresponding number of amino acids in GGS repeats (α^ΔCyt61aa^ and β^ΔCyt48aa^), and mutants with swapped cytoplasmic domains (α^βWT^ and β^αWT^). Quickchange site-directed mutagenesis kit (Agilent Technologies) was used to create tyrosine-to-phenylalanine mutations of the Igα-ITAM (tyrosines 182 and 193). The resulting constructs were cloned into pcDNA6 vector (Clontech) and were transiently expressed in primary B cell blasts using the Amaxa B cell nucleofector kit (Lonza). Transfected B cells were maintained in RPMI1640 supplemented with 10% FCS, 2 mM Glutamine, 50 µM β-mercaptoethanol and 1% ITS (Sigma) and used 24 hours post-transfection. To express Igα and Igβ constructs in HEK293A cells, the suspension cells were transfected along with the nitrophenyl-specific B1-8 Igµ and Igλ, with linear polyethylenimine (l-PEI, MW∼25, 000, Polysciences) as described [30]. Briefly, a transfection mix of 2 µg/ml plasmid DNA and 4 µg/ml l-PEI in OptiMEM medium (Invitrogen) were first incubated at room temperature for 10 minutes and then added to 1×106/ml HEK293A cells in suspension. Transfected HEK293A cells were used on day 3 post-transfection.

### Imaging And Image Analysis

Cells were resuspended in Hank's balanced salt solution (HBSS) supplemented with 0.1% BSA and were attached to poly-L-lysine-coated coverslip chambers (Labtek). For CFP quenching, a final concentration of 10 µM R18 (Molecular Probes) was added to cells after the first timepoint, followed by gentle mixing to allow rapid and simultaneous labelling of cells. We found that R18 photobleaching could not be used to measure FRET, because of high-rate of R18 photoconversion into the CFP channel. Photobleaching of CFP was minimal under these conditions. For BCR stimulation of R18 labeled cells, 10 µg/ml goat F(ab′)_2_ anti-mouse IgM (Jackson Immunoresearch) was added to cells. For inhibitor studies, 50 µM PP2 (Sigma) was added to cells for 30 minutes at 37°C before stimulation. Live-cell imaging was carried out at room temperature on an Olympus IX81 microscope, with an Andor iXon EMCCD Camera and 100× objective (Olympus). Epifluorescence timelapse images of individual cells were acquired every 2 minutes for 10 min. CFP fluorescence was excited using a 442 nm laser and collected using a 480/40 nm emission filter. FRET channel used the same excitation, but collected R18 fluorescence using 585/70 nm emission filter. R18 fluorescence was excited using a 514 nm laser and collected using a 585/70 nm emission filter.

All images were analyzed using ImageJ. Fluorescence was measured as average pixel intensity from regions of interest covering the plasma membrane. Background was subtracted from regions of interest in unlabeled cells. FRET efficiency using CFP quenching was determined by percentage of decrease in CFP intensity after addition of R18. Controls showed that there were no changes in the CFP channel in cells not expressing CFP. FRET ratio was determined as (F - γ*A)/D, where F, A and D are the intensities in the FRET, acceptor (R18) and donor (CFP) channels, respectively. γ is a correction factor defined by F/A from R18-labeled cells, which do not express CFP.
